# Syntaxin-1 and Insulinoma-Associated Protein 1 Expression in Breast Neoplasms with Neuroendocrine Features

**DOI:** 10.3389/pore.2021.1610039

**Published:** 2021-10-26

**Authors:** Sándor Turkevi-Nagy, Ágnes Báthori, János Böcz, László Krenács, Gábor Cserni, Bence Kővári

**Affiliations:** ^1^ Department of Pathology, University of Szeged, Szeged, Hungary; ^2^ Laboratory of Tumor Pathology and Molecular Diagnostics, Szeged, Hungary; ^3^ Bács-Kiskun County Teaching Hospital, Kecskemét, Hungary; ^4^ Department of Anatomic Pathology, H. Lee Moffitt Cancer Center & Research Institute, Tampa, FL, United States

**Keywords:** immunohistochemistry, syntaxin-1, insulinoma-associated protein 1, breast neuroendocrine neoplasms, solid papillary carcinoma, mucinous carcinoma, carcinoma no special type

## Abstract

**Introduction:** A subset of breast neoplasia is characterized by features of neuroendocrine differentiation. Positivity for Neuroendocrine markers by immunohistochemistry is required for the diagnosis. Sensitivity and specificity of currently used markers are limited; based on the definitions of WHO Classification of Tumours, 5th edition, about 50% of breast tumors with features of neuroendocrine differentiation express chromogranin-A and 16% express synaptophysin. We assessed the applicability of two novel markers, syntaxin-1 and insulinoma-associated protein 1 (INSM1) in breast carcinomas.

**Methods:** Hypercellular (Type B) mucinous carcinomas, solid papillary carcinomas, invasive carcinomas of no special type with neuroendocrine features and ductal carcinomas *in situ* of neuroendocrine subtype were included in our study. The immunohistochemical panel included chromogranin A, synaptophysin, CD56, syntaxin-1 and INSM1. The specificity of syntaxin-1 and INSM1 was determined using samples negative for chromogranin A, synaptophysin and CD56.

**Results:** The sensitivity of syntaxin-1 was 84.7% (50/59), with diffuse positivity in more than 60% of the cases. Syntaxin-1 also had an excellent specificity (98.1%). Depending on the definition for positivity, the sensitivity of INSM1 was 89.8% (53/59) or 86.4% (51/59), its specificity being 57.4% or 88.9%. The sensitivities of chromogranin A, synaptophysin and CD56 were 98.3, 74.6 and 22.4%, respectively.

**Discussion:** Syntaxin-1 and INSM1 are sensitive and specific markers of breast tumors with neuroendocrine features, outperforming chromogranin A and CD56. We recommend syntaxin-1 and INSM1 to be included in the routine neuroendocrine immunohistochemical panel.

## Introduction

It has been known for decades, that a subset of breast neoplasia may present with either histomorphological or immunohistochemical (IHC) signs of neuroendocrine (NE) differentiation, or a combination thereof (1). Although these features were primarily described in hypercellular (Type B) mucinous carcinomas, it has become evident that solid papillary carcinomas and many other invasive breast carcinomas of no special type also exhibit such a phenotype (2–5).

NE differentiation, as a histological type defining criterion, was introduced only in the third edition of the World Health Organization (WHO) classification (“blue book” series) of breast tumors (6). This edition, separately from the mucinous carcinomas, mentioned the NE tumors as a distinct category with subcategories: solid, small cell/oat cell and large cell NE carcinomas. These entities were defined on the one hand by the histomorphologic similarities with the NE neoplasms of other organs (e.g., the gastrointestinal tract and lungs), on the other hand by immunoreactivity for NE markers in at least 50% of the tumor cells. Although this category was mainly defined by morphological features, it was not clearly separated from other special types of breast carcinomas known for frequent expression of NE markers. Since its introduction, the classification, definitions and taxonomy have undergone several modifications.

The fourth edition refined the diagnostic criteria by omitting the 50% threshold of immunoreactivity and, separately from “pure” NE tumors and carcinomas, also introduced a new class, namely invasive breast carcinoma with NE differentiation (7). Large cell NE carcinoma was excluded from this edition.

Currently, the fifth and latest classification, harmonized with the newest consensus proposal of the International Agency for Research on Cancer (IARC) and the WHO, adopted the term “neuroendocrine neoplasm” (NEN) (8, 9). It includes well-differentiated NENs also known as NE tumors (NETs) and poorly differentiated NENs or (small cell and large cell) NE carcinomas (NECs). Solid papillary carcinoma and the hypercellular variant (Type B) of mucinous carcinoma remained as distinct entities with frequent NE differentiation. Even though this system seems to have separated the pure NENs from other entities, some of the remaining, much more common categories are unfortunately not so well delineated. If a tumor displays histological features and immunoreactivity for NE markers, but is not “distinct or uniform enough”, the appropriate diagnosis should be invasive carcinoma no special type with NE differentiation. Furthermore, if a conventional neoplasm contains areas (between 10 and 90%) consistent with NEN, the term “mixed NEN” should be used.

Besides histomorphology, IHC evaluation is required for confirming NE differentiation. The most commonly applied NE markers are chromogranin A (CGA), synaptophysin (SYP) and CD56. However, none of these markers is sensitive and specific enough to be used alone, and consensus reports recommend their combined application. At the same time, novel, more sensitive or specific and more easily applicable NE marker candidates are being identified. Beside insulinoma-associated protein 1 (INSM1), another example for such a promising molecule is syntaxin-1 (STX1), which we reported to be a generally reliable NE marker (10).

INSM1 is a transcription factor which takes part in the development of NE tissues and neoplasia. Beside regulating transcription, INSM1 is also crucial in arresting cell cycle, therefore it is considered as a key molecule in terminal NE differentiation (11). STX1 is an essential molecule of the neurosecretory machinery and acts as a component of the SNARE complex. Its role is to enable fusion of the secretory vesicle and the presynaptic membrane. Apart from neurons, STX1 has been proven to be specifically expressed by NE cells.

The aim of our study was to evaluate the characteristics of STX1 and INSM1 IHC expression in breast neoplasia showing NE features.

## Materials and Methods

Formalin-fixed paraffin-embedded samples were collected from the archives of the Departments of Pathology of the Bács-Kiskun County Teaching Hospital and the University of Szeged. Diagnoses of all cases were updated according to the criteria of the 5th edition of WHO classification of breast tumors (8). NE differentiation was defined as immunoreactivity with at least one classical NE marker (CGA, SYP or CD56). However, only tumors raising the possibility of NE differentiation were stained for these markers during the routine work-up.

To evaluate the samples, tissue microarrays (TMAs) were used. The TMA blocks were constructed manually as previously published (12–14). Briefly, cores of 2.2 mm in diameter were sampled from both the periphery and the centre of the lesions; each lesion being represented in either two or three cores.

Three to four-micrometer-thick sections were used for IHC reactions with STX1, CGA, SYP, CD56 and INSM1 antibodies, in all lesions. Primary antibodies and the applied protocols are listed in Table 1 and have been reported in detail previously (10).

**TABLE 1:** Primary antibodies and IHC protocols.

Antibody	Clone	Manufacturer	Retrieval	Dilution
STX1 (HPC-1)	sc-12736 (Mouse monoclonal)	Santa Cruz	pH 10.0	1:200
INSM1	A8 (Mouse monoclonal)	Santa Cruz	pH 9.0	1:100
SYP	27G12 (Mouse monoclonal)	Novocastra	pH 9.0	1:400
CGA	LK2H10 (Mouse monoclonal)	Cellmarque	pH 9.0	1:700
CD56	123C3.D5 (Mouse monoclonal)	Cellmarque	pH 9.0	1:200

Altogether, 113 cases (79 from the archives of the Bács-Kiskun County Teaching Hospital, the remaining 34 from the University of Szeged) diagnosed between 2001 and 2019 were collected. Fifty-nine tumors from 55 patients (4 of them with bifocal lesions) demonstrated traditional NE marker positivity and the remaining 54 were negative for these markers and formed a negative control group in our study. All lesions with NE marker positivity were diagnosed either as hypercellular (Type B) mucinous carcinoma, solid papillary carcinoma, invasive breast carcinoma of no special type with NE features or ductal carcinoma *in situ*, NE subtype. No tumor in this series fulfilled the criteria of NET or NEC. The included cases are briefly summarized in Table 2.

**TABLE 2:** Tumor types of the included cases.

Institute	Diagnosis	No of cases
Bács-Kiskun County Teaching Hospital	Mucinous carcinoma, Type B	10
	Solid papillary carcinoma	12
	Invasive carcinoma NST with NE differentiation	11
	DCIS, NE subtype	1
University of Szeged	Mucinous carcinoma, Type B	12
	Solid papillary carcinoma	11
	Invasive carcinoma NST with NE differentiation	1
	DCIS, NE subtype	1
		59
	Non-NE cases for analysis of specificity	54
Altogether		113

NST, invasive carcinoma of no special type; DCIS, ductal *in situ* carcinoma.

**FIGURE 1:**
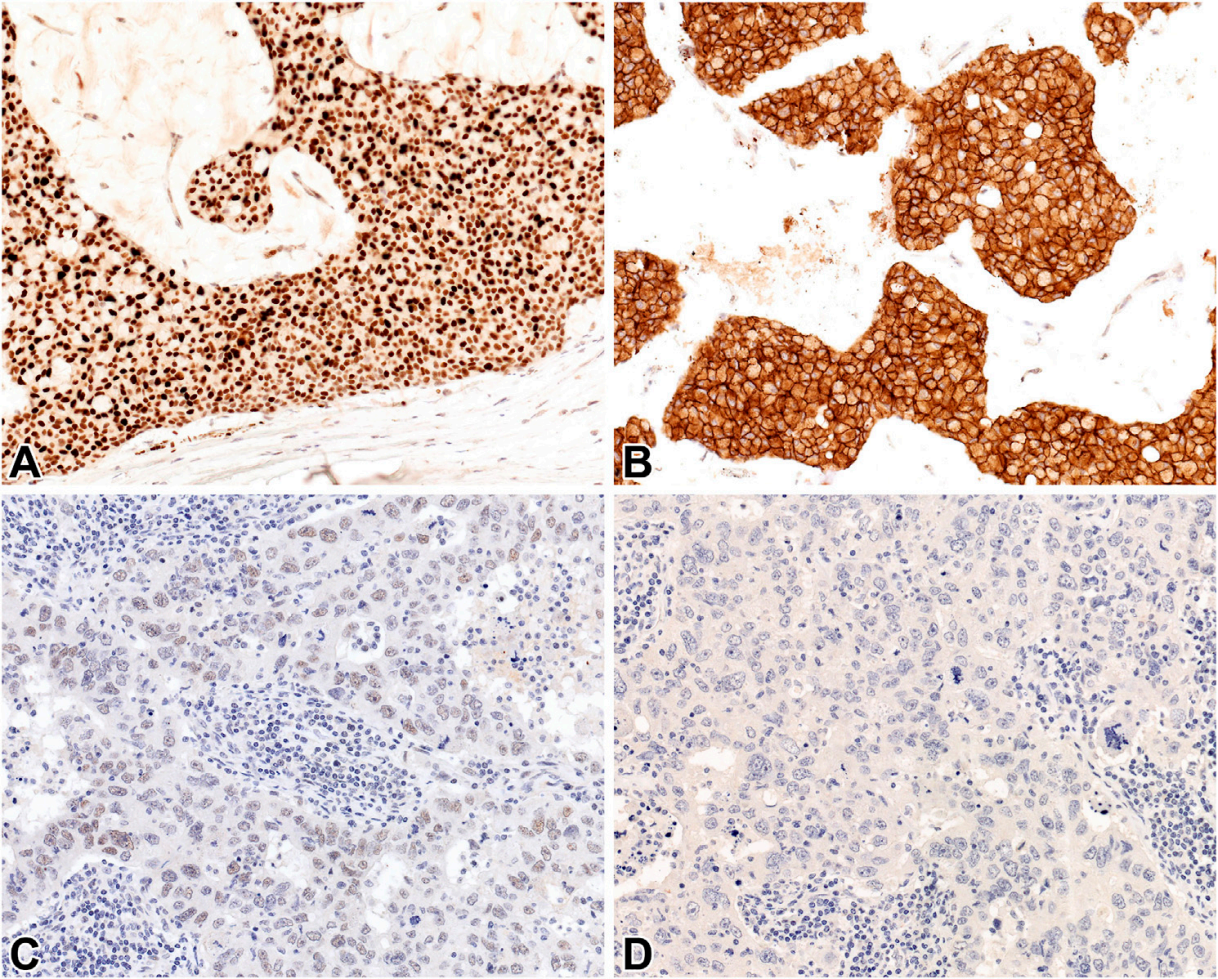
INSM1 **(A, C)** and STX1 **(B, D)** immunoreactivity in a hypercellular (Type B) mucinous carcinoma **(A, B)** and invasive carcinoma NST without NE features **(C, D)** 20x.

For CGA and SYP, any (at least 1%) cytoplasmic labelling of the tumor cells; for INSM1, any (at least 1%) nuclear positivity; finally, for STX1 and CD56, any (at least 1%) cytoplasmic and/or membranous staining were considered positive. The percentage of the labelled tumor cells, as well as the semiquantitative (0 to 3+, respectively) intensity of the staining was evaluated separately by 3 pathologists. Subsequently, consensus at a multiheaded microscope was reached for discrepant cases. Based on a frequently detected focal and weak INSM1 expression in a pilot series, INSM1-stained slides were also evaluated using two additional practical definitions for positivity; 1) any nuclear staining of any intensity [referred to as high-power (HP) positivity], 2) nuclear staining obvious even at low-power view [referred to as low power (LP) positivity] (Figure 1).

To assess the specificity of the novel markers, STX1 and INSM1 IHC reactions were performed on samples derived from other breast carcinomas proven to be negative for CGA, SYP and CD56 (Table 2). For this purpose, TMA technique was applied as well.

No patient-related information was collected; materials were collected anonymously and retrospectively with no influence on outcome or treatment. The study was approved by the Clinical Research Coordination Office of the University of Szeged (4430/2018).

## Results

**FIGURE 2:**
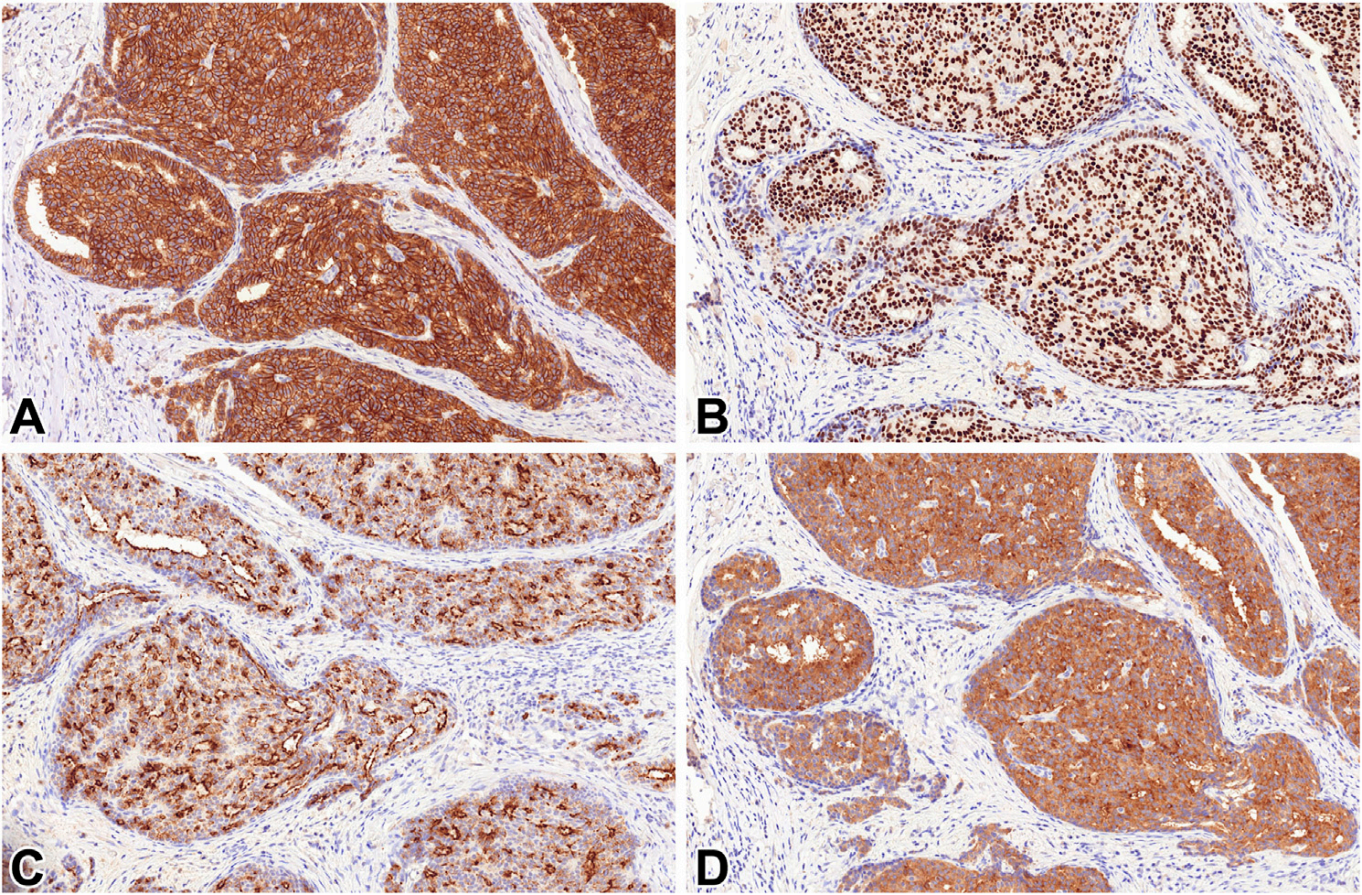
STX1 **(A)**, INSM1 **(B)**, CGA **(C)** and SYP **(D)** immunoreactivity in a solid papillary carcinoma 20x.

STX1 immunoreactivity was detected in 50/59 tumors. The labelling was diffuse in 37 (62.7%) of the 59 lesions. The median percentages of positive tumor cells were 85 and 55% for cytoplasmic or membranous staining patterns, respectively (Figure 2).

INSM1 expression was noted in 53/59 lesions on HP and 51/59 on LP, with a uniform nuclear pattern. Independently of the applied threshold, positivity was diffuse in 28/59 (47.5%) lesions, while the median percentage of labelled tumor cells was 50%.

Regarding the classical NE markers, the ratios of the positive cases and the median percentages of positive tumor cells were 58/59 and 80% for SYP, 44/59 and 50% for CGA and 13/58 and 0% for CD56, respectively. Diffuse positivity was present in 69.5% (41/59) for SYP, 47.5% (28/59) for CGA and 5.2% (3/58) for CD56.

The overall sensitivities of the novel markers were 89.8 and 86.4% for INSM1 on HP and LP, respectively, and 84.7% for STX1. Concerning the classical NE molecules, the sensitivities were 98.3% for SYP, 74.6% for CGA and 22.4% for CD56. The median intensity of staining was strong (3+) for each observed marker, with the exception of CD56 (2+). The data and descriptive values are summarized in Tables 3, 4.

**TABLE 3:** Characteristics and sensitivity of STX1, INSM1, SYP, CGA and CD56 IHC in lesions with NE features.

	STX1	INSM1		SYP	CGA	CD56
		HPF	LPF			
Positive/observed cases	50/59	53/59	51/59	58/59	44/59	13/58
Median % of labelled cells	85% c 55% m	50%	50%	80%	50%	0%
Median % of labelled cells in positive cases	90% c 75% m	55%	55%	82.5%	72.5%	25%
Diffusely positive cases/observed cases (%)	28/59 (47.5%)	37/59 (62.7%)	37/59 (62.7%)	41/59 (69.5%)	28/59 (47.5%)	3/58 (5.2%)
Sensitivity	84.7%	89.8%	86.4%	98.3%	74.6%	22.4%
(95% CI)	(0.725–924)	(0.785–0.958)	(0.745–0.936)	(0.897–0.999)	(0.613–0.846)	(0.129–0.356)

c, cytoplasmic; m, membranous; HPF, high power field; LPF, low power field.

**TABLE 4:** Ranges of cases according to the percentage of labelled cells.

		% of labelled cells			
		0–25	26–50	51–75	76–100
STX1	C	17	6	3	33
	M	22	6	7	22
INSM1	LPF	20	11	7	21
	HPF	20	11	7	21
SYP	-	17	1	6	35
CGA	-	24	7	6	22
CD56	-	52	3	1	2

c, cytoplasmic; m, membranous; HPF, high power field; LPF, low power field.

Regarding the specificity of the novel markers, only a single STX1 positive case was detected in the negative control group (1/54), resulting in a specificity of 98.1%. As for INSM1, some cases exhibited very faint and generally focal staining which was obviously present at HP (×40 objective) magnification, but was not seen or much less obvious on LP (×4 objective) examination (Figure 1). Applying the HP threshold, 23/54 cases were found to be positive while the specificity was 57.4%; however, using LP, the ratio of positive cases was only 6/54, increasing the specificity of INSM1 significantly, to 88.9%. Specificity of the novel markers is outlined in Table 5.

**TABLE 5:** Specificity of STX1 and INSM1.

	STX1	INSM1	
		HPF	LPF
Negative/observed cases	53/54	31/54	48/54
Specificity	98.1%	57.4%	88.9%
(95% CI)	(0.888–0.999)	(0.433–0.705)	(0.767–0.954)

HPF, high power field; LPF, low power field.

## Discussion

The classification of breast lesions with NE features or differentiation had undergone several changes and refinements since its introduction but has still not reached an easily and consistently usable state. Due to the variable and sometimes obscure definitions and classifications as well as the lack of routine IHC examination of NE marker expression, the reported incidence (ranging between 0.1 and 20%) of breast tumors showing NE features is likely unreliable (15). These factors may contribute to the fact that the prognostic significance of NE differentiation in breast tumors is still somewhat uncertain.

In spite of the continuously changing definitions and thresholds, immunoreactivity with NE markers seems to be a constant requirement, and as such, a critical step to make the diagnosis. IHC for the demonstration of several markers has long been applied for this purpose. However, no single marker is known to be sensitive and specific enough to be used on its own. To overcome this challenge, numerous efforts are made to identify other suitable candidates. Conceptually, these novel biomolecules may either serve as components of the neurosecretory apparatus, or act as master regulators of NE differentiation. The latter category is represented by INSM1, a transcription factor which is of particular interest and has been a subject of recent studies in various organs (16–19). An example for the former one is STX1, which has also proved to be a sensitive and specific NE marker (10).

The aim of this study was to assess the applicability of STX1 and INSM1 as NE markers of breast lesions, as well as to compare their performance with the traditional molecules used to assess NE differentiation (i.e., SYP, CGA and CD56). Similarly to results from other organs, STX1 proved to be a reliable marker for the diagnosis of NE breast lesions, with a sensitivity of 84.7%, characterized by a convincing, diffuse immunoreactivity in 62.7% of the cases included. A strong and easy-to-read membranous labelling pattern was also noted beside cytoplasmic staining in the majority of the lesions. Our experience was that the nuclear staining pattern of INSM1 (similarly to the membranous STX1-labelling) was more convenient to interpret than the cytoplasmic expression pattern of other markers. The different subcellular locations of INSM1 and STX1 labelling enable the use of double immunohistochemical staining method in biopsy cases with limited neoplastic tissue. Apart from exhibiting great sensitivity, STX1 was also characterized by an excellent specificity (98.1%), with clear-cut negativity in all but one sample in the control group.

In the case of INSM1, sensitivity was excellent without significant difference between LP and HP definition for positivity (89.8 and 86.4%, respectively); but more than half of the evaluated lesions showed only focal positivity. However, the negativity of the control cases was rather equivocal. If the more permissive LP definition was applied, the specificity of INSM1 was found to be only 57.4%; however, when the HP definition was used, it increased to 88.9%. This observation supports the findings of a recent study of more than one thousand breast carcinomas, in which “a slightly higher cut-off for a positive result” was determined for INSM1 in order to reach a specificity of 98.1% (17). The fact that 17 tumors without NE features in our series would have been misclassified depending on the threshold, raises some concerns regarding the specificity of INSM1. However, given the excellent sensitivity, INSM1 is strongly recommended to be used in combination with other, more specific markers.

As concerns the traditional NE markers, the greatest sensitivity was achieved with SYP (98.3%), nevertheless, STX1 mildly outperformed it in the median percentage of labelled cells (STX1: 85% vs. SYP: 80%). The remaining two classical NE molecules, CGA and especially CD56 exhibited unexpectedly low sensitivities (74.6% for CGA and 22.4% for CD56). Our experience with the latter marker is comparable to the findings of a recent publication (16), therefore CD56 should probably be decommissioned from the general NE marker arsenal, at least in the setting of breast tumors.

There were obvious limitations and unexplained aspects in the present study. A fraction of the investigated tumors showing INSM1 expression only using the HP definition exhibited immunoreactivity against INSM1 in the absence of every other markers. This phenomenon, which was also observed by other authors, raises the concerns regarding the specificity of INSM1 even further. In a recent study by Zombori et al, focal INSM1 IHC expression was also detected in non-NE pulmonary carcinomas (19). The isolated positivity for INSM1 without detectable expression of other components of the neurosecretory apparatus may be explained by an interrupted cascade of yet-to-be-found intermediate mediators, which transfer the signal of NE differentiation from the transcription factor. This would make INSM1 expression a necessary, but, on its own insufficient condition for this lineage of development (20).

Another possible explanation is the immanent focality of NE differentiation in the majority of breast tumors. Apart from intra-tumoral heterogeneity, these neoplasms are diverse per diagnostic categories, too. It is known that only around 50% of solid papillary carcinomas are positive for NE markers (20), which may be a plausible explanation for the anomaly. Given these facts, despite multiple sampling, the TMA method may have led to false negative results. Furthermore, the retrospective nature of the study and the low number of included cases may have biased our findings. However, NE markers are also needed in the routine reporting of core biopsies that are likewise subject to intra-tumoral heterogeneity; thus, data obtained using the TMA technique may be used to extrapolate how these markers would perform in the core biopsy setting. Altogether, we believed that the few dozens of cases investigated were sufficient to validate the concept of STX1 and to a lesser extent of INSM1 as suitable NE markers of breast tumors, even if we were unable to formulate statements concerning the exceptionally rare primary mammary NETs or NECs.

In conclusion, consistently with data from other organs, we propose that STX1 is a promising novel, highly sensitive and specific, easily applicable NE marker. Along with INSM1, another recently identified and already better studied (16, 18, 20) molecule, we strongly recommend STX1 to be included in the routine diagnostic IHC panel of NE differentiation. Following further studies, STX1 and INSM1 may become ancillary markers of SYP and be able to replace the less sensitive CGA and CD56 in the area of breast neoplasia with NE features.

## Data Availability

The raw data supporting the conclusions of this article will be made available by the authors, without undue reservation.
